# Omics characteristics and epigenetic modifications of adipose-derived stem cells

**DOI:** 10.1016/j.jbc.2025.110758

**Published:** 2025-09-24

**Authors:** Tiange Feng, Clifford J. Rosen, Ziru Li

**Affiliations:** 1 MaineHeath Institute for Research, Center for Molecular Medicine, Scarborough, Maine, USA; 2 Department of Medicine, Tufts University School of Medicine, Boston, Massachusetts, USA

**Keywords:** adipose-derived stem cells, omics, epigenetic modifications, adipogenesis, osteogenesis

## Abstract

Adipose-derived stem cells (ADSCs) possess multipotency to differentiate into various lineages, including adipocytes, osteoblasts, neurons, and smooth muscle cells, thereby demonstrating significant potential in tissue regeneration. Recent advances in omics technologies have enhanced our understanding of ADSC molecular profiles by transcriptomic, proteomic and lipidomic analysis. Additionally, epigenetic modifications, which mediate heritable changes in gene expression without altering the DNA sequence, play a pivotal role during the multiple lineage differentiation of ADSC. This review presents a summary of recent findings on ADSC omics and epigenetics, offering an updated perspective on their distinct attributes.

Multipotent stem cells are characterized by their capacity for self-renewal and their ability to differentiate into a limited range of cell types within a specific lineage (1). Among these, adipose-derived stem cells (ADSCs) are particularly notable for their extensive differentiation potential, which encompasses adipogenesis, osteogenesis, chondrogenesis, myogenesis, angiogenesis, cardiomyogenesis, tenogenesis, and periodontogenesis (2). Beyond their cellular plasticity, ADSCs secrete a diverse array of bioactive molecules that confer immunoregulatory, proangiogenic, and neurotrophic effects, underpinning their robust regenerative capabilities (3). A key advantage of ADSCs over other stem cell sources is their accessibility; they can be easily isolated in large quantities through minimally invasive procedures such as liposuction or needle biopsy from peripheral adipose tissue (4). Collectively, these attributes position ADSCs as an exceptionally promising and practical resource for a spectrum of applications in regenerative medicine. To effectively harness this therapeutic potential for safe and predictable clinical outcomes, however, a foundational understanding of their biology is imperative.

Therefore, a comprehensive understanding of core ADSC features, including their gene expression profiles, epigenetic modifications, and differentiation potential, is crucial (Fig. 1). This review synthesizes recent advancements from omics studies (transcriptomics, proteomics, and lipidomics) and epigenetic analyses that illuminate the molecular mechanisms governing ADSC differentiation and functionality.

**Figure 1:**
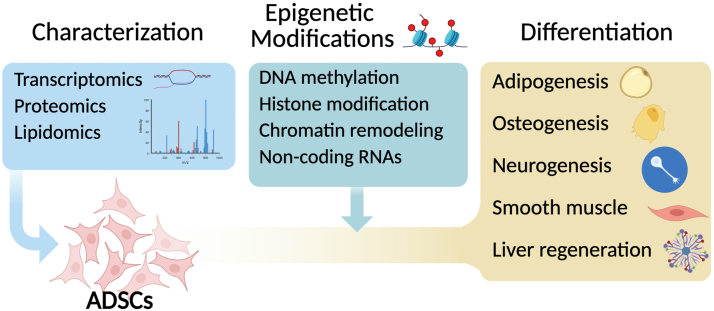
**This review covers a range of topics, from omics studies characterizing adipose-derived stem cells (ADSCs) to the influence of epigenetic modifications on ADSC lineage differentiation (Created in**https://BioRender.com).

## Omics-based analyses characterize ADSC features in transcriptome, proteome, and lipidome

The omics studies hold the premise to advance the accuracy and comprehensiveness of our understanding of ADSC functions and characteristics. For instance, transcriptomic profiling could reveal active gene expression and cell population heterogeneity through bulk and single-cell level RNA sequencing. Proteomic analysis of ADSCs offers another dimension for characterizing ADSC features at a translational level. Since adipocytes are unique in their lipid storage and utilization, lipidomics on ADSCs and differentiated mature adipocytes may provide novel insights into the lipid species that regulate their distinct functions.

### Transcriptomics

The transcriptomic landscape of ADSCs has been investigated *via* high-throughput RNA sequencing since 2014 (5), with a recent emphasis on achieving single-cell resolution. Subsequent analyses of white adipose tissue (WAT) using single-cell and single-nucleus RNA sequencing (scRNA-seq and snRNA-seq) have been pivotal in uncovering considerable heterogeneity within ADSC populations, both in their marker gene expression profiles and their lineage commitment capabilities (6, 7, 8). One ADSC subcluster, with a high degree of expression of adipogenic markers such as *Pparg* and *Cd36,* demonstrates a high capacity for adipogenesis and is considered committed preadipocytes (9). Another fraction of adipose stem and precursor cells is characterized by high *Cd142* and *Abcg1* expression and negatively regulates the adipogenic capacity of other progenitor cells *via* paracrine mechanisms, including secretion of SPINK2, VIT and/or FGF12 (10). IL-33^+^ ADSC subgroup is positively correlated with enriched regulatory T (Treg) cells in the epididymal WAT of male mice under physiologic conditions (11); these Treg cells facilitate the process of constraining local inflammation in adipose tissue and may potentially benefit insulin sensitivity (12). Distinct from the previous review that thoroughly detailed the classification and functions of ADSC subpopulations (9), this discussion focuses on comparing ADSCs with stem cells from other tissues and elucidating obesity and sex-dependent distinctions observed in both mice and humans.

Comparative analysis between ADSCs and multipotent stem cells originating from other tissues identifies both shared and unique characteristics of these cell populations. Roson-Burgo *et al.* compared the microarray-based transcriptomic data from human ADSCs (hADSCs), bone marrow-derived mesenchymal stem cells (BMSCs), and placenta-derived stem cells, as well as fibroblasts from dermal tissue in a meta-analysis. All cell populations share a common mesenchymal lineage expression core signature, such as stromal cell biomarkers *THY1* (*CD90*) and *NT5E* (*CD73*), transcription factor *SNAI2* (a regulator of epithelial-mesenchymal transition during embryonic development), and several collagen molecules (*COL3A1, COL4A1, COL4A2, COL5A1, COL5A2, COL6A3,* and *COL12A1*). Whereas compared with other stem cell sources, ADSCs exhibited the fewest unique gene expression profiles, suggesting that this cell population may need fewer specific genes to perform its functions or is more committed to particular lineage(s) (13). Similarly, another microarray-based comparison of the transcriptomes of subcutaneous ADSCs and BMSCs from castrated Yorkshire crossbred male pigs revealed that differentially upregulated genes in ADSCs were predominantly enriched in myogenic-related functions, including *FN1*, *SPP1*, and *VEGFA*. In contrast, BMSCs exhibited a greater number of upregulated genes related to angiogenic, osteogenic, cell migration and adhesion processes (14).

The transcriptome of ADSCs can be significantly altered under obese conditions. Subcutaneous ADSCs from morbidly obese patients presented marked upregulation of genes promoting adipocyte lineage commitment and pro-inflammatory signaling compared to those from non-obese individuals. While ADSCs from obese patients retain similar stem cell surface marker profiles, they show significant transcriptional changes in genes associated with cell survival, differentiation, tissue integrity, and metabolic regulation (15).

ADSCs also exhibit distinct transcriptomics profiles based on sex. For instance, studies on male mouse perigonadal WAT ADSCs reveal elevated expression of *Hox(abc)1 to 8* transcripts, while those from female mice display increased levels of *Hox(acd)9 to 13* (16). Given that *Hox* genes are critical determinants of segment identity during metazoan embryonic development (17), these heterogeneous expression patterns may indicate partially divergent developmental trajectories of perigonadal WAT in males and females. Such differences could, in turn, underpin variations in adipocyte function, tissue remodeling, and inflammatory responses between sexes. For example, male mouse ADSCs demonstrate enhanced signaling through the Renin-Angiotensin-Aldosterone System (RAAS), a pathway implicated in inflammation, glucose metabolism disorders, and the hemodynamic regulation of WAT (16). A separate bioinformatics analysis of human subcutaneous ADSCs, integrating multiple microarray datasets, also unveiled sex-related differences (18). Male hADSC samples exhibited significant upregulation of integrin subunits (*ITGB8, ITGA8*, and *ITGB3*) compared to female samples. These integrins are crucial for cell adhesion, intercellular signaling, stem cell migration, and anchoring within the cellular niche (19, 20). Conversely, genes highly expressed in female hADSCs included *ANKK1* and *CXCL3*, which involves in neural development (18) and inflammatory responses (21, 22), respectively.

Notably, the upregulation of integrin genes in male hADSCs in the human study aligns conceptually with the murine findings regarding *Hox* gene expression. Both gene families play vital roles during adipose tissue development: *Hox* genes regulate adipogenesis *via* PPARγ/retinoic acid receptor (RAR)-dependent pathways in mouse preadipocytes (23), and integrins are essential for extracellular matrix reorganization, maintaining WAT insulin sensitivity, and regulating substrate transport (24). Despite these underlying commonalities in developmental significance, the specific differentially expressed gene profiles identified in the two studies are not identical. This discrepancy likely stems from limitations in microarray technology regarding the spectrum of detectable genes (18) and the inherent differences in ADSC sources between the murine perigonadal and the human subcutaneous WAT.

### Proteomics

Mass spectrometry–based proteomic approaches have been employed to identify ADSC-specific markers that distinguish them from other cell types based on tissue or species origin, exosomal cargo, and culture conditions.

In human ADSCs and BMSCs, intraindividual comparative proteomic analysis following 21 days of osteogenic induction identified 2095 commonly expressed proteins. Notably, 427 proteins were uniquely detected in ADSCs in over 80% of patient samples. These ADSC-enriched proteins were predominantly associated with biological oxidation, nucleobase biosynthesis, and vitamin and cofactor metabolism. In contrast, BMSCs exhibited elevated expression of proteins involved in extracellular matrix (ECM) organization and cell–matrix interactions. These findings suggest that ADSCs exhibit higher basal metabolic activity, whereas BMSCs display more pronounced capacity of osteogenic differentiation and bone matrix production (25).

Notably, exosomes from different stem cell sources carry distinct sets of enriched cargo proteins that closely mirror the proteomic profiles of their cells of origin. BMSC-derived exosomes are enriched in proteins associated with collagen synthesis, ECM organization, bone regeneration, and muscle repair. ADSC exosome cargos exhibit prominent secretory functions and play roles in immune modulation. Whereas umbilical cord MSC exosomes show superior efficacy in tissue injury repair. Collectively, the cargo proteins within stem cell–derived exosomes reflect the dominant signaling pathways active in their respective source cells (26).

Interestingly, ADSCs continue to reflect a high level of metabolic activity even under hypoxic conditions (1% O_2_). In porcine ADSCs, hypoxia induces the upregulation of numerous glycolysis-related proteins, including ALDOC, TPI1, GAPDH, PGK1, PGAM1, ENO1, ENO2, and LDHA, indicating enhanced anaerobic energy metabolism. Additionally, the cells exhibit molecular adaptations to the low-oxygen environment, with increased expression of proteins involved in intracellular communication and mechanotransduction pathways, such as MAPK1, ZYX, and VCL (27).

### Lipidomics

The types and composition of lipid species within cells are pivotal regulatory factors of cellular functions and the stem/progenitor cell fate (28). Current studies on lipid profiling in ADSCs remain largely descriptive, with limited investigation into the functional roles of specific lipid species.

In an untargeted lipidomics analysis of human peripheral blood mononuclear cells (PBMCs), human fibroblasts and hADSC, phospholipids and sphingomyelins (SMs) were the lipid classes detected across all samples. A distinctive and more diverse phospholipid profile has been observed in ADSCs, with specific species such as phosphatidylglycerol (PG) 40:7 and phosphatidylethanolamine (PE) O-36:3 detected exclusively in ADSCs, but absent in PBMCs and fibroblasts (29). In another study that specifically targeted sphingolipids in human MSCs derived from bone marrow, adipose tissue, and umbilical cord tissue, SMs exist predominantly in ADSCs (30). These findings suggest a potential role for phospholipids and sphingolipids in regulating ADSC function; yet their specific effects remain unexplored.

In a study analyzing the lipidomic difference between rabbit ADSCs and skeletal muscle-derived stem cells (MDSCs) from rabbits, samples were collected under undifferentiated, adipogenic and osteogenic conditions (28). Among the identified 1687 lipid species, 27 significantly different lipid species were identified between all groups of ADSCs and MDSCs. In undifferentiated ADSCs, the relative concentrations of N-acyl-phosphatidylethanolamine (NAPE) 54:7 (containing 54 carbon atoms and seven double bonds), NAPE 56:0, phosphatidylserine (PS) 34:1 and phosphatidylethanolamine (PE) 32:1 were higher compared to all other groups (28). NAPE species represent a minority class of phospholipids, involved in regulating several physiological processes, including inflammation, cell division and embryogenesis (31). PS lipids are found in cellular membrane that enhance membrane integrity and neurotransmitter release (32). PE acts as a molecular “chaperone” to assist membrane proteins in achieving correct tertiary structure for proper functionality. It also plays roles in autophagy, membrane fusion, and mitochondrial biogenesis (33). Further investigation of their roles in ADSCs is warranted.

### Summary

While various omics studies reveal fundamental common and distinct features between ADSCs and other stem cells, most studies report divergent molecular signatures with minimal overlap. This inconsistency partly reflects the cells' responsiveness to environmental cues and their intrinsic properties, as well as differences in research focus, species, and experimental conditions across studies (Table 1). It also underscores the current lack of cross-platform comparison and mechanistic integration among high-throughput omics analyses of ADSCs.

**Table 1:** Omics-based analyses characterizing ADSC features

Omics	Species	Approach	Main finding(s)	Reference
Transcriptomics	Human/mouse	scRNA-seq and snRNA-seq	There is considerable heterogeneity within ADSC populations	(6, 7, 8, 9, 10, 11)
	Human	Microarray	ADSCs share some common stem cell signatures but possess a unique transcriptional profile	(13)
	Porcine	Microarray	ADSCs exhibited a predominant enrichment in myogenic-related genes compared to BMSCs	(14)
	Mouse	scRNA-seq	ADSCs exhibit sex-specific transcriptomic differences, with male ADSCs enriched for inflammatory and glucose metabolism disorder-related signatures	(16) (18)
	Human	Microarray		
Proteomics	Human	Proteomics-osteogenesis	ADSCs exhibit higher basal metabolic activity	(25)
	Human	Exosome cargo proteomics	ADSCs possess prominent secretory functions and contribute to immune modulation	(26)
	Porcine	Proteomics-hypoxia condition	ADSCs adapt to hypoxia with enhanced anaerobic energy metabolism	(27)
Lipidomics	Human	Untargeted lipidomics	A distinctive and more diverse phospholipid profile has been observed in ADSCs	(29)
	Human	Targeted sphingolipids	Sphingomyelins enrich in ADSCs	(30)
	Rabbit	Untargeted lipidomics	ADSCs are enriched in specific NAPE, PS and PE species	(28)

## Epigenetic regulations in ADSCs

Epigenetics involves heritable changes in gene expression and long-term alterations in transcriptional potential that do not alter the underlying DNA sequence (34). These regulatory mechanisms include DNA methylation, histone modifications, and chromatin remodeling. Additionally, regulatory non-coding RNAs (ncRNAs), especially microRNA (miRNA), play a significant role in epigenetic regulation and are often considered an integral part of the broader epigenetic machinery (35). These modifications are crucial in regulating gene expression and cellular differentiation in stem cells, supporting their proliferation and maturation into specialized cell types (36). Although comprehensive reviews of mesenchymal stem cell epigenetics exist (35), to date, there has been no literature summarizing advances in this field focused specifically on ADSCs. Therefore, we sought to provide a targeted description of ADSC epigenetic modifications and consequential influence on cell differentiation, especially on adipogenesis and osteogenesis.

## Epigenetic control of ADSC adipogenesis

The adipogenic potential of ADSCs is closely associated with their epigenetic modifications, which can either promote or inhibit adipogenesis depending on the context of specific factors, highlighting a complex regulatory network.

### DNA methylation

Yoshitaka Kubota *et al.* identified two distinct adipose progenitor cell populations: ceiling culture-derived preadipocytes (ccdPAs) and ADSCs. Upon adipogenic induction, ccdPAs demonstrated a higher capacity for differentiation compared to ADSCs. Genome-wide analyses revealed that ccdPAs exhibited lower levels of promoter CpG methylation and increased enrichment of trimethylated histone H3 at lysine 4 (H3K4me3) at key adipogenic loci, including *Pparg* (peroxisome proliferator-activated receptor γ), *Fabp4* (fatty acid-binding protein 4), and *Lep* (leptin) (37). These findings suggest that ccdPAs are more committed to adipogenic lineage differentiation, a process associated with reduced CpG methylation. Transcription factors and other regulatory molecules that modulate DNA methylation can indirectly influence the adipogenic commitment of ADSCs. For example, zinc-finger proteins can recognize methylated DNA (38), specifically binding to CpG sites and thereby modulating the differentiation potential of these stem cells. A recent study demonstrated that overexpression of Zinc Finger Protein 521 (ZNF521) in human ADSCs significantly delays and reduces adipocyte differentiation, at least in part due to ZNF521's inhibition of a transcription factor, early B-cell factor 1 (EBF1) (39), which interacts with ten-eleven translocation-2 (TET2) to promote DNA demethylation (40). Conversely, silencing ZNF521 markedly enhances adipogenic differentiation (39).

Systemic metabolic homeostasis alterations, including conditions like obesity and diabetes, are capable of influencing ADSC adipogenic potential through modifications to their DNA methylation profile. Studies comparing hADSCs from visceral adipose tissue of obese patients, with or without type 2 diabetes (T2D), have revealed distinct DNA methylation patterns during different stages of adipogenic differentiation (stem cell, preadipocyte, and mature adipocyte). Cells from T2D patients displayed epigenetic changes across all differentiation stages compared to non-diabetic individuals, with alterations affecting genes linked to adipogenesis, insulin resistance, cell death, and immune processes. These findings suggest the presence of an epigenetic “metabolic memory” associated with T2D (41). Moreover, hypertrophic WAT, typically correlated with insulin resistance and T2D, also exhibits unique epigenetic signatures. Increased CpG methylation was observed in hypertrophic WAT compared to hyperplastic WAT, with 98% of CpG sites displaying consistent changes in genes related to insulin resistance, lipolysis, extracellular matrix organization, and innate immunity (42). In the context of obesity, nuclear-encoded mitochondrial gene methylation is also modified and contribute to the dysfunction of ADSCs. In a study comparing ADSCs from the abdominal subcutaneous fat of obese and non-obese individuals, hydroxymethylated DNA immunoprecipitation sequencing (hMeDIP-seq) and mRNA sequencing identified a select group of nuclear-encoded mitochondrial genes involved in ATP production, redox activity, cell proliferation, migration, fatty acid metabolism, and neuronal development (43). These findings suggest that epigenetic modifications of nuclear-encoded mitochondrial genes are associated with mitochondrial dysfunction in obese human ADSCs.

### Histone modification and chromatin interactions

Regulation of various functional molecules in histone modification pathways or alterations in chromatin conformation also influence ADSC adipogenesis. For example, histone 2B (H2B) O-GlcNAcylation mediates the positive effects of AMPKα1 on the expression of isocitrate dehydrogenase 2 (IDH2), which is crucial for brown adipogenesis (44). O-GlcNAcylation, a nutrient-sensitive modification catalyzed by O-linked N-acetylglucosamine transferase (OGT), involves the addition of a single GlcNAc moiety to serine or threonine residues of target proteins, playing a role in the epigenetic regulation of gene expression (45). Decreased H2B O-GlcNAcylation and monoubiquitination levels were associated with reduced *Idh2* expression in brown adipose tissue stromal vascular cells isolated from AMPKα1 knockout mice (44). Additionally, the histone acetylase inhibitor, C646, blocked cell proliferation, arrested the cell cycle, and induced apoptosis. However, it unexpectedly led to an increase in histone H3K9 acetylation and the upregulation of two other acetylases, TIP60 and PCAF. As a result, C646 treatment significantly promoted adipogenic differentiation (46).

Differential histone modifications at gene promoters influence depot-selective gene expression of ADSCs, reported by a comparative study of ADSCs derived from abdominal *versus* gluteofemoral fat depots between individuals with apple- and pear-shaped obesity (47). To investigate the role of histone modifications in depot-selective gene expression, a comparative study was conducted in individuals with apple- and pear-shaped obesity to examine the correlation between histone markers (H3K4me3, H3K4me2, H3K27me3, and H3K9me3) and differential gene expression in ADSCs derived from abdominal *versus* gluteofemoral fat depots (47). Their analysis suggested that histone modifications influenced the patterns of depot-selective expression of genes involved in both adipogenesis, adipose tissue expansion, and intriguingly, genes that limit adipogenesis (*INHBA*, *TIMP1*, and *CDKN2B*) (47). However, it is unclear whether differential gene expression is the cause or consequence of histone modification.

Of note, traditional approaches to exploring post-transcriptional regulation often relied on correlating differential gene expression with the linear distance of genes on chromatin (48). However, this method is limited by the fact that eukaryotic chromatin is organized into a three-dimensional, topologically constrained network, where distal regulatory elements can be in spatial proximity to gene promoters, which cannot be predicted based on linear genomic distance alone. The study mentioned above (47) also identified remote genomic regions influencing depot-specific gene regulation in ADSCs isolated from abdominal and gluteofemoral regions. There were 35 unique differentially expressed genes (DEGs) that were potentially regulated through the enhancer loop interaction, and some likely influence differential fat distribution (*HOXA*, *BDNF*, *IL33*, *EPHX2* and *IGF2BP1*). This study revealed the complex epigenetic regulation of depot-specific gene expression in these stem cells.

### microRNAs

The regulation of ADSC adipogenic differentiation is significantly influenced by various microRNAs, which modulate key signaling pathways and transcriptional regulators. For instance, miR-27b and miR-138 inhibit adipogenic differentiation of hADSCs by targeting lipoprotein lipase (LPL) (49, 50). Dual luciferase reporter assays confirmed the negative regulatory interaction between miR-27b/miR-138 and LPL. Overexpression of miR-27b or miR-138, or LPL knockdown, resulted in reduced levels of key adipogenic transcription factors, such as CCAAT-enhancer binding protein α (C/EBPα) and PPARγ (49, 50). Moreover, increased expression of miR-27a-3p and miR-27b-3p are involved in the anti-adipogenic effects of 1,25-dihydroxyvitamin D_3_ in both 3T3-L1 cells and human ADSCs (51). MiR-138 inhibits adipogenesis *via* early region 1-A-like inhibitor of differentiation 1 (EID-1), a nuclear receptor coregulator. The expression of EID-1 was inversely correlated with miR-138 levels, and EID-1 knockdown similarly inhibited adipogenesis. Luciferase assays confirmed that miR-138 directly targets the 3′ untranslated region of EID-1. These findings suggest that miR-138 inhibits adipogenic differentiation, in part, by repressing both LPL and EID-1, highlighting the broader role of miRNAs in regulating hADSC differentiation (52).

Inversely, miR-30c enhances human adipocyte differentiation by co-repressing PAI-1 (encoded by *SERPINE1*) and ALK2 (encoded by*ACVR1*). MiR-30c expression was significantly upregulated during adipogenic differentiation of hADSCs. Overexpression of miR-30c promoted the induction of adipocyte-specific marker genes and increased triglyceride accumulation. Simultaneous silencing of both PAI-1 and ALK2 recapitulated the pro-adipogenic effects observed with miR-30c overexpression, suggesting that miR-30c promotes adipogenesis by repressing these key inhibitory regulators (53).

In summary (Table 2), most nuclear and mitochondrial DNA methylation events exert negative effects on ADSC adipogenesis. A similar trend is observed in histone modifications. In contrast, miRNAs exhibit diverse effects depending on their target genes. However, due to the limited available literature and the complex nature of epigenetic modifications, it remains challenging to draw definitive conclusions. Comprehensive profiling approaches, such as ChIP assays and ATAC-seq, may offer a more detailed landscape of these DNA modifications and their influence on adipogenic gene expression.

**Table 2:** Epigenetic regulators in ADSC adipogenesis

Regulator Category	Specific Regulator(s)	Effect on adipogenesis	Mechanism(s)	Reference
DNA Methylation	H3K4me3	Promote	Co-occurs with lower CpG methylation	(37)
	ZNF521	Inhibit	Inhibits transcription factor EBF1/TET2 → Promote DNA methylation	(39)
	TET enzymes	Promote	Hydroxylase activity promotes DNA demethylation	(40)
	Mitochondrial DNA Methylation	Inhibit/Dysregulate	Contributing to mitochondrial dysfunction	(43)
Histone Modification	Histone 2B (H2B) O-GlcNAcylation	Promote	Mediates positive effects of AMPKα1 on IDH2 expression	(44)
	Histone acetylase inhibitor C646	Promote	Upregulates the expression of acetylases, TIP60 and PCAF	(46)
	Chromatin Conformation	Promote/Inhibit (Depot-Specific)	Chromatin loops can link enhancers physically close to their target genes	(47)
microRNAs (miRNAs)	miR-27a-3p, miR-27b	Inhibit	Suppresses LPL mRNA	(49, 51)
	miR-138	Inhibit	Represses both LPL and EID−1	(50, 52)
	miR-30c	Promote	Co-represses PAI−1 and ALK2	(53)

## Epigenetic control of ADSC osteogenesis

### DNA methylation

The dynamic interplay between DNA methylation and demethylation is crucial during development, establishing distinct and stable DNA methylation patterns that regulate tissue-specific gene transcription as cells differentiate (54). The TET proteins oxidize 5-methylcytosine (5mC) to form 5-hydroxymethylcytosine (5hmC), 5-formylcytosine (5fC), and 5-carboxylcytosine (5caC), which are key intermediates in the DNA demethylation process (44). Studies have indicated that increased expression of TET genes and elevated levels of 5hmC are critical for osteogenic differentiation of ADSCs. During ADSC osteogenesis, key epigenetic alterations include the loss of 5mC and the corresponding increase in 5hmC, align with the upregulation of TET1 expression (55). Moreover, TET2 is upregulated during hADSC osteogenesis, while inhibition of TET2 reduces osteogenic-related gene expression and 5hmC levels, potentially compromising bone health and contributing to osteoporosis. Indeed, TET2-deficient mice exhibit significant reductions in femoral length, bone mineral density, and bone volume fraction, underscoring the importance of TET2 in maintaining bone integrity (56).

The diabetic environment impairs the osteogenic potential of ADSCs isolated from inguinal fat through DNA methylation. Zhang *et al.* found that ADSCs from diabetic osteoporosis (DOP) mice exhibited reduced osteogenic capacity and diminished Wnt/β-catenin signaling, which correlated with a elevated level of DNA methyltransferase-3a (Dnmt3a). Moreover, downregulation of Dnmt3a rescued the poor osteogenic potential of DOP-ADSCs by enhancing osteogenic factor expression and reactivating Wnt/β-catenin signaling, while Dnmt3a overexpression inhibited this process (57). DOP-ADSCs also exhibit decreased expression of LncRNA-AK137033, which further inhibits the Wnt signaling pathway by modulating the DNA methylation level in the promoter region of the *sFrp2* gene (58).

Age is another factor that influences osteogenesis through epigenetic mechanisms. ADSCs derived from older donors (>60 years) possess markedly reduced osteogenic differentiation potential and lower 5hmC levels compared to those from younger individuals (<45 years). Treatment with the DNA demethylating agent 5-azacytidine enhanced osteogenic differentiation in aged ADSCs by promoting cell proliferation and increasing alkaline phosphatase activity, a crucial factor for bone homeostasis. This improvement in osteogenesis is linked to elevated 5hmC levels and the upregulation of TET2 and TET3 gene expression (59).

### Histone modification

A histone methyltransferase, enhancer of Zeste homolog 2 (EZH2), responsible for the trimethylation of histone H3 at lysine 27 (H3K27me3), was found to inhibit osteogenic differentiation. High-throughput mRNA sequencing revealed that inhibiting EZH2 activates cell cycle inhibitory proteins and increases the production of extracellular matrix components (60).

Histone acetylation, in contrast, promotes ADSC osteogenesis. For instance, histone H3K18 acetylation (H3K18ac) is significantly upregulated during osteogenic differentiation, with this modification being enriched in the promoter regions of key osteogenic genes. Histone acetyltransferase p300 serves as the enzyme responsible for this process, as its inhibition with A-485 reduced H3K18ac levels and impaired osteoblast function (61). In alignment with this, inhibition of histone deacetylase (HDAC) also enhances the osteogenic differentiation of human ADSCs *in vitro*. Wu *et al.* demonstrated that pre-treatment of hADSCs with the HDAC inhibitor MI192 significantly promoted osteogenic differentiation, as evidenced by increased expression of key osteogenic markers such as runt-related transcription factor 2 (RUNX2), collagen type 1 (COL1), and osteocalcin (OCN), along with elevated alkaline phosphatase activity and matrix mineralization (62).

Mechanical stimulation also induces epigenetic modifications that accelerate osteogenic differentiation in ADSCs. Daily mechanical stretching of human ADSCs over 7- and 15- days led to significant downregulation of DNA methylation at critical CpG sites within the NESP and GNASXL promoter regions. This reduction in methylation was associated with an earlier initiation of osteogenic differentiation (63). Similarly, Ma *et al.* screened histone demethylase expression in rat ADSCs under varying matrix stiffness and identified histone lysine demethylase 3B (KDM3B) as a positive regulator of osteogenesis. On stiff matrices, ADSCs adopted a widespread, polygonal morphology with organized vinculin, unlike the rounded, disorganized appearance on soft matrices. Stiff polydimethylsiloxane also upregulated osteogenic markers, including *Ocn*, alkaline phosphatase (*Alpl*) and *Runx2* in rat ADSCs. Inversely, *Kdm3b* knockdown reversed these effects, decreasing osteogenic markers and impairing mitochondrial dynamics and membrane potential. These findings underscore KDM3B's crucial role in biomechanics-driven ADSC osteogenic commitment (64).

Topography regulates histone modification and cell fate during differentiation. TiO2 nanotubes promote osteogenic differentiation of hADSCs by enhancing the histone H3 at lysine 4 (H3K4) methylation level at the promoter regions of osteogenesis-related genes, and upregulating lysine demethylase 4E (KDM4E), which catalyzes demethylation of H3K9 (65). In addition, TiO-nanotube also downregulates HDACs, therefore, enhances acylation levels of histones (65). Generally, higher methylation levels of H3K4, lower methylation level of H3K9, and histone acetylation lead to transcriptional activation (66).

### Non-coding RNAs

The regulatory role of ncRNAs in osteogenesis depends on the functions of their target molecules during ADSC osteogenic differentiation. Hydrostatic pressure (HP) enhances osteogenesis of hADSCs by increasing lncRNA-PAGBC (prognosis-associated gallbladder cancer lncRNA), which acts as a competitive endogenous RNA (ceRNA) by binding to the osteogenesis-inhibitory microRNA, miR-133b (67). *RUNX2*, a key gene in osteogenesis, is a target of miR-133b. Overexpression of lncRNA-PAGBC increased *RUNX2* expression, while lncRNA-PAGBC silencing decreased *RUNX2* levels through the miR-133b (67).

miR-375 promotes the osteogenic differentiation of hADSCs through the YAP1/DEPTOR/AKT regulatory network. Mechanistically, miR-375 downregulates DEPTOR, thereby activating Akt signaling and osteogenic differentiation. Additionally, miR-375 targets YAP1, which in turn binds to the miR-375 promoter to inhibit its expression, forming a feedback loop that modulates osteogenesis (68). Similarly, miR-4699 promotes osteogenic lineage commitment of hADSCs by suppressing the expression of *DKK1* and *TNFSF11* genes. Overexpression of miR-4699 stimulates key osteoblast markers including *RUNX2*, *ALPL*, and *OCN*, as well as enhances alkaline phosphatase activity and matrix mineralization (69). Additionally, during hADSC osteogenic differentiation, endogenous expression of miR-26a increases significantly. Overexpression of miR-26a enhances osteogenesis, whereas its knockdown inhibits this process. MiR-26a directly targets the 3′ UTR of *GSK3β*, leading to a reduction in GSK3β protein levels and an increase in osteogenic differentiation (70). Moreover, miR-196a overexpression reduced the proliferation of hADSCs while enhancing osteogenic differentiation without impacting adipogenic differentiation (71). The pro-osteogenic effects may partially act through decreasing the protein and mRNA levels of *HOXC8*, a predicted target of miR-196a (71).

Meanwhile, miR-100 has been identified as a negative regulator of the osteogenic differentiation in hADSCs through its targeting of bone morphogenetic protein receptor type II (BMPR2). *In vitro* studies demonstrated that overexpression of miR-100 inhibited osteogenic differentiation, while its downregulation promoted this process. Target prediction analysis and dual luciferase reporter assays confirmed that *BMPR2* is a direct target of miR-100. The knockdown of *BMPR2* *via* RNA interference resulted in inhibited osteogenic differentiation of hADSCs, mirroring the effects observed with elevated levels of miR-100 (72). miR-24-3p shares the same osteogenic inhibitory effect by directly binding to the 3′UTR of tribbles pseudokinase 3 (*TRIB3*) gene to block its expression. TRIB3 enhances alkaline phosphatase activity, matrix mineralization, and heterotopic bone formation (73). miR-137 negatively regulates osteogenesis by directly targeting the *NOTCH1* gene and establishing a complex co-regulatory network involving NOTCH1, LSD1, and BMP2, all of which are crucial signaling molecules for osteogenesis (74). In addition, miR-137 directly targets and downregulates *LGR4* expression, while stimulating *RANKL* expression, together exerting a coordinated inhibitory effect on osteogenesis (75).

Similar to the epigenetic modifications observed in adipogenesis, DNA and histone methylation generally inhibits, while DNA demethylation and histone acylation promote, osteogenesis. These processes can be modulated by enzymatic activity, diabetic conditions, aging, mechanical stimuli, and topographical cues (Table 3).

**Table 3:** Epigenetic regulators in ADSC osteogenesis

Regulator Category	Specific Regulator(s)	Effect on osteogenesis	Mechanism(s)	Reference
DNA Methylation	TET Proteins	Promotes	Initiate DNA demethylation, increasing 5hmC levels	(55, 56)
	Dnmt3a	Inhibits	Suppresses Wnt/β-catenin signaling	(57)
	Decrease of lncRNA-AK137033	Inhibits	Reduces the DNA methylation level in the *sFrp2* promoter region	(58)
	5-azacytidine	Promotes	DNA demethylating agent, upregulating TET2 and TET3 expression	(59)
Histone Modification	EZH2	Inhibits	Catalyzes the formation of the repressive histone mark H3K27me3	(60)
	H3K18 acetylation	Promotes	Histone acetyltransferase p300 increases H3K18ac in the promoter regions of key osteogenic genes	(61)
	HDAC inhibitor MI192	Promotes	Inhibits histone deacetylase to enhance osteogenic gene expression	(62)
	Mechanical Stimulation	Promotes	Downregulates DNA methylation at critical CpG sites	(63)
	Matrix Stiffness	Promotes	Promotes KDM3B activity to enhance osteogenesis and mitochondrial dynamics	(64)
	TiO_2_ Nanotubes (Topography)	Promotes	(1) Activates H3K4 methylation, (2) demethylate the repressive H3K9 mark, and (3) increases histone acetylation	(65)
Non-coding RNAs	lncRNA-PAGBC	Promotes	An RNA sponge for miR-133b, preventing its inhibition on *Runx2*	(67)
	miR-375	Promotes	Downregulates *DEPTOR* to activate pro-osteogenic Akt signaling	(68)
	miR-4699	Promotes	Suppresses *DKK1* and *TNFSF11* expression	(69)
	miR-26a	Promotes	Targets the 3′ UTR of *GSK3β* and reduces its protein levels	(70)
	miR-196a	Promotes	May act through decreasing *HOXC8* expression	(71)
	miR-100	Inhibits	Directly targets and represses *BMPR2* mRNA	(72)
	miR-24-3p	Inhibits	Suppresses *TRIB3* gene expression	(73)
	miR-137	Inhibits	Targets and downregulates *NOTCH1* and *LGR4*, while enhances *RANKL* expression	(74, 75)

## MicroRNAs that simultaneously affect ADSC adipogenesis and osteogenesis

Notably, epigenetic regulation mediated by certain microRNAs influences both differentiation pathways simultaneously. For instance, miR-22 plays a dual role by promoting osteogenic differentiation while inhibiting adipogenesis in hADSCs. Overexpression of miR-22 in hADMSCs inhibits lipid droplet accumulation and downregulates adipogenic transcription factors and adipocyte-specific genes. Conversely, enhanced alkaline phosphatase activity, matrix mineralization, and elevated osteogenic marker gene expression following miR-22 overexpression indicate a stimulatory role in osteogenesis (76).

miR-21 expression is elevated during both adipogenesis and osteogenesis in hADSCs, correlating with increased differentiation potential. Upregulation of miR-21 enhances ERK-MAPK activity, a crucial regulator that promotes hADSC differentiation, while downregulation reduces it during the early stages of differentiation. miR-21 exerts this influence by repressing SPRY2, a key regulator of receptor tyrosine kinase (RTK) signaling, thereby modulating the amplitude and duration of ERK-MAPK signaling during hADSC differentiation (77).

Both miR-17-5p and miR-106a play crucial roles in lineage commitment of hADMSCs by directly targeting BMP2. Their activity leads to the suppression of osteogenic markers such as TAZ, MSX2, and Runx2, while simultaneously upregulating adipogenic markers including C/EBPα and PPARγ (78).

## Epigenetic modifications on other differentiation pathways and functions of ADSCs

### Neurogenic differentiation

ADSCs also possess the epigenetic predisposition toward neural differentiation, a pre-patterning that is further enhanced by the demethylation of specific Nestin (*NES*) enhancer elements following mitogenic stimulation prior to neurogenic differentiation. Nestin, a marker highly expressed in neural progenitors, is upregulated in ADSCs following mitogenic stimulation, mirroring its behavior in neural progenitors, and is subsequently downregulated during the neurogenic differentiation of ADSCs. The neural-specific enhancer regions of the *NES* gene undergo DNA demethylation upon mitogenic stimulation but are re-methylated during the neurogenic differentiation process. Concurrently, dynamic modifications in histone marks, such as H3K4, H3K9, and H3K27 methylation, are observed at the *NES* locus, aligning with the epigenetic regulation of *NES* expression (79). Significant increases in histone three trimethylation (H3K4me3, H3K9me3, H3K27me3) and HDAC activities are also observed during spheroid formation of hADSCs for treating nerve injuries. Specifically, H3K4me3 (KMT2A) and H3K9me3 (SUV39H1) are involved in the transient state of spheroid formation. Moreover, H3K9me3 and HDAC5 play major regulatory roles in the end stage of spheroid formation and neural differentiation in hADSCs (80). Moreover, Hernandez *et al.* identified significant changes in the DNA methylation of six genes, hypomethylation (*Hoxa-5, GRM4, FGFR1*) and hypermethylation (*RTEL1, METRN, PAX9*), during neuronal differentiation in hADSCs. Among them, *Hoxa-5* overexpression was able to induce neuronal differentiation (81). The findings highlight the crucial role of these epigenetic factors in shaping stem cell fate and guiding lineage-specific differentiation.

In a study that investigates the role of microRNAs in promoting the neurogenic differentiation of ADSCs, miR-124 was found significantly upregulated during this process. The knockdown of miR-124 impairs both neurogenic differentiation and the electrophysiological characteristics acquired by the cells. miR-124 directly targets *RHOA* (Ras homolog gene family member A) mRNA, repressing its expression, which in turn increases the proportion of neurogenic transdifferentiation markers such as *NSE* (neuron-specific enolase), *Tuj-1* (Neuron-specific class III beta-tubulin) and *GFAP* (glial fibrillary acidic protein) (82).

In addition, ADSCs could be differentiated into Schwann-like cells to promote the repair of peripheral nerve injury. Overexpression of *Gdnf* (glial cell line-derived neurotrophic factor) in ADSCs increased the expression of the Schwann cell marker S100, and other neurotrophic factors, such as CNTF, BDNF, NT3, GFAP and GAP43. Mechanistically, *Gdnf* overexpression upregulated *Mta1* (metastasis-associated gene 1) expression in ADSCs, which positively regulates the downstream *Hes1* molecule expression promoting differentiation into Schwann cells (83). This may promote peripheral nerve regeneration following injury, though *in vivo* validation is still required.

### Smooth muscle differentiation

Single-cell RNA sequencing and trajectory analysis revealed the differentiation potential of perivascular adipose tissue-derived ADSCs (PV-ADSCs) to smooth muscle lineage. This was exemplified by a transplantation of cultured PV-ADSCs in a mouse vein graft model, where PV-ADSCs contribute to vascular remodeling partly through smooth muscle cell (SMC) differentiation. Mechanistically, treatment with transforming growth factor β1 (TGF-β1) and transfection of miR-378a-3p mimics induced similar metabolic reprogramming in PV-ADSCs, including elevated mitochondrial potential and altered lipid profiles, such as increased cholesterol levels (84).

Another study found that miRNA-145 enhances the differentiation of hADSCs into SMCs by downregulating *KLF4* (Krüppel-like factor 4). Levels of *KLF4* declined during the differentiation of hADSCs and were further downregulated when the cells were transfected with miR-145 mimics. Additionally, inhibiting *KLF4* through treatment with short-interfering RNA (siRNA) led to an upregulation of SMC-specific genes and proteins (85).

### Liver regeneration

ADSCs also possess the potency to differentiate into hepatocyte-like cells *in vitro*, hosting functional properties of primary human hepatocytes, such as gene expression of *Alb* (albumin), *Tdo2* (tryptophan 2,3-dioxygenase) and *Hnf-3b* (hepatocyte nuclear factor 3b), after differentiating for 13 days (86). Transplantation of ADSC-derived hepatocyte-like cells into CCl_4_-induced liver injury model facilitated liver regeneration and decreased circulating markers of liver injury, including alanine aminotrans-ferase, aspartate aminotransferase, as well as ammonia (86). Hepatic differentiation of hADSCs could be negatively regulated by let-7f miRNA. When hADSCs were transfected with a recombinant lentivirus containing the human let-7f inhibitor, gene expression of hepatocyte markers and nuclear factors were upregulated, including hepatocyte nuclear factors alpha, albumin, alpha fetoprotein, cytokeratin 18, and cytokeratin 19 (87).

Besides potential contribution to hepatocytes, ADSCs also inhibit the activation, proliferation, and function of immune cells (88). In a study involving rats subjected to 70% partial hepatectomy, overexpression of miR-27b in ADSCs significantly enhanced liver regeneration, promoted hepatic differentiation, and suppressed liver inflammation and fibrotic activity (89). Although the molecular mechanisms were not well-defined in this study, it is possible that factors released by miR-27b transfected ADSCs are also responsible for the reduction of inflammatory response and myofibroblast differentiation in the resected liver.

### Immunomodulation

The immunomodulatory function of MSCs is partly attributed to the expression of human leukocyte antigen (HLA)-G (90). In a recent study, adipose- and bone marrow-derived stem cells were treated with DNA demethylating agent 5-aza-2-deoxycytidine (5-aza-dC) and histone deacetylase inhibitor valproic acid (VPA) to assess their impact on HLA-G expression. Treatment with 5-aza-dC led to the upregulation of HLA-G1 and HLA-G3 mRNA in both early and late passage MSCs, while VPA treatment had no effect on HLA-G expression in either adipose- or bone marrow-derived MSCs. This study provides the first evidence of HLA-G3 expression in MSCs and marks DNA methylation's role in suppressing HLA-G expression. These findings indicate that epigenetic modulators, such as 5-aza-dC, may enhance the immunoregulatory capabilities of MSCs, offering potential strategies for improving their therapeutic applications (91).

In summary (Table 4), ADSCs not only possess the potential to differentiate into neural cells, SMCs, and hepatocyte-like cells, but also contribute to neuroprotection through Schwann-like cell differentiation and play immunomodulatory roles in the context of tissue injury or cell transplantation, highlighting their translational clinical potential.

**Table 4:** Epigenetic regulations of other ADSC differentiation pathways

Lineage	Potential Regulator(s)	Reference
Neurogenesis	Histone mark demethylation and methylation at the Nestin locus	(79)
	Histone 3 trimethylation and HDAC activities enhance ADSC spheroid formation and neural differentiation	(80)
	*Hoxa-5* induces neuronal differentiation	(81)
	MiR-124 represses *RHOA* expression and promotes neurogenic transdifferentiation	(82)
	*Gdnf* upregulates *Mta1* that promotes ADSC differentiation into Schwann cells	(83)
Smooth muscle cell	TGF-β1 and miR-378a-3p mimics induce metabolic reprogramming	(84)
	MiRNA-145 enhances the differentiation of ADSCs into SMCs by downregulating *KLF4*	(85)
Hepatocyte-like cell	Let-7f miRNA negatively regulates hepatic differentiation	(87)
	MiR-27b promotes hepatic differentiation and suppresses inflammation	(89)

## Epigenetic modifications in senescence and apoptosis of ADSCs

Aging and functional changes pose significant challenges to the *in vitro* culture and clinical application of ADSCs, with epigenetic modifications playing a crucial regulatory role in these processes.

### DNA methylation

Culture expansion of ADSCs induces highly reproducible DNA methylation changes, associated with repressive histone marks, specifically the loss of H3K4me3 and the presence of H3K9me3 (92). Although karyotyping and SNP-microarray analysis detected no chromosomal aberrations, the frequency of fibroblastoid colony-forming units (CFU-f) and the osteogenic and adipogenic differentiation potential of ADSCs showed a substantial decline over passages. These alterations were accompanied by consistent senescence-associated modifications at specific CpG sites (93).

Interestingly, treatment with DNA methyltransferase (DNMT) inhibitors, such as 5-Azacytidine (5-AZA), has been shown to reverse the aged phenotype of human subcutaneous ADSCs. The application of 5-AZA increased the proliferation rate, decreased oxidative stress factors and DNA methylation levels, and elevated the mRNA expression of TET proteins involved in the demethylation process. Additionally, cells treated with 5-AZA exhibited reduced reactive oxygen species (ROS) accumulation, enhanced superoxide dismutase activity, and an increased BCL-2/BAX ratio (indicating a decreased susceptibility to apoptosis) compared to controls (94), which may contribute to enhanced cell survival and overall anti-aging effects. Additionally, hypoxic preconditioning has shown beneficial effects on swine ADSCs derived from models of atherosclerotic renal artery stenosis (ARAS). Compared with healthy controls, ARAS ADSCs exhibited impaired angiogenesis, reduced proliferative and migratory capacity, and increased senescence, accompanied by elevated baseline levels of specific histone modifications and DNA hydroxymethylation. Hypoxic preconditioning mitigated these dysfunctions by restoring proliferative and migratory potential, reducing senescence, enhancing pro-angiogenic gene expression, and lowering global 5hmC levels (95). Collectively, these findings demonstrate that ADSCs undergo significant epigenetic alterations, including histone modifications and DNA methylation, during senescence. Targeting these changes may represent a promising strategy to optimize the therapeutic applications of ADSCs.

### Non-coding RNAs

miR-486-5p induces replicative senescence in hADSCs. Its overexpression leads to a premature senescence-like phenotype, inhibiting the proliferation and differentiation (both adipogenic and osteogenic). In contrast, the inhibition of miR-486-5p has opposite effects, promoting proliferation and differentiation. SIRT1 (Silent information regulator 1) is one of miR-486-5p′s targets, and a key regulator of longevity and metabolic disorders. Notably, a decrease in SIRT1 deacetylase activity in hADSCs correlates with their passage number (96). Similarly, the inhibition of miR-34a has been shown to reduce cellular senescence in hADSCs by activating SIRT1. Silencing miR-34a enhances proliferation, promotes adipogenic and osteogenic differentiation, reduces senescence-associated β-galactosidase activity, and reverses molecular alterations associated with senescence in hADSCs (97).

Additionally, miR-301a significantly suppressed the gene expression of apoptosis signal-regulating kinase 1 (ASK1), which has been found to attenuate the apoptosis of hADSCs transplanted into infarcted heart tissue. MiR-301a-enriched hADSCs exposed to hypoxic conditions exhibited a significant down-regulation of apoptosis-related genes. These findings suggest that the miR-301a-mediated down-regulation of ASK1 protects ADSCs during post-transplantation, potentially enhancing the efficacy of ADSC-based cell therapy (98).

Although DNA methylation—particularly at specific CpG sites—has significant effects on maintaining ADSC stemness, differentiation, and viability, the use of DNA methylation inhibitors and demethylation activators offers promising strategies to overcome these limitations. Various miRNAs play distinct roles in regulating cell senescence, underscoring the complexity of non-coding RNA biology and the need for further investigation. Additionally, interactions among different miRNAs remain poorly understood and warrant more in-depth study.

## Summary and discussion

**Figure 2:**
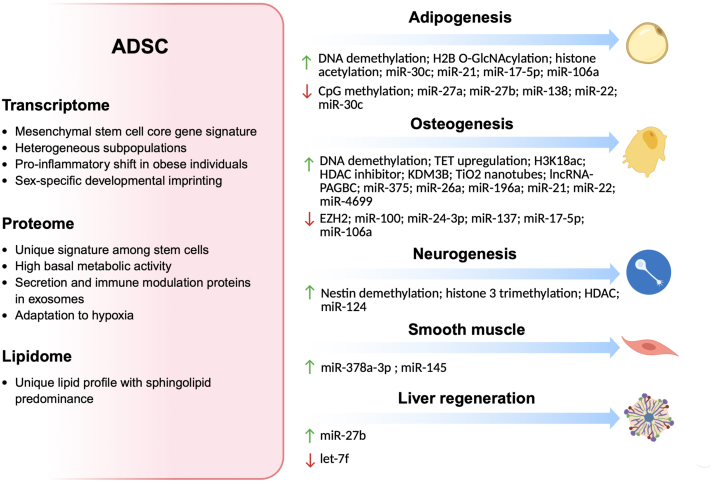
**Multi-omics characteristics of ADSCs and epigenetic regulation factors of differentiation.***Green upward arrows* represent promoting effects; *red downward arrows* represent inhibitory effects (Created in https://BioRender.com).

Recent advances in omics and epigenetics research emphasize that the remarkable plasticity of ADSCs is not merely a passive potential, but is actively and dynamically regulated by a complex network of epigenetic mechanisms. This regulatory system—including DNA methylation, diverse histone modifications, and a wide array of non-coding RNAs—functions as a sophisticated integrative hub, translating both intrinsic cellular programs and extrinsic signals from the micro- and macro-environment into specific lineage outcomes. This review highlights that ADSC fate is highly context-dependent, shaped by factors such as donor age, metabolic status (*e.g.*, obesity and type 2 diabetes), fat depot of origin, and exposure to physical or chemical stimuli (Fig. 2). Importantly, many epigenetic regulators function as molecular switches—for instance, miR-22 has been shown to promote osteogenesis while inhibiting adipogenesis—underscoring the tightly orchestrated nature of lineage specification.

These findings summarized herein have important implications for ADSC-based clinical applications. In particular, they may inform strategies to enhance cell stability and therapeutic performance by modulating epigenetic determinants. At the same time, the review identifies emerging challenges, such as cellular senescence during *in vitro* expansion, which may compromise regenerative potential. As the field progresses beyond descriptive profiling of epigenetic modifications, new mechanistic questions and technological demands are rapidly coming into focus.

### Emerging challenges in the field

Despite rapid progress, significant hurdles remain that limit the clinical translation of ADSC therapies. Firstly, many studies demonstrate a strong correlation between a specific epigenetic mark and a differentiation outcome. However, proving direct causality remains a major challenge. For example, does the loss of H3K27me3 at a gene promoter cause its activation, or is it a consequence of other upstream events that activate transcription? Without establishing causality, targeting specific epigenetic modifiers for therapy is fraught with uncertainty. Secondly, it has been technically challenging to bridge the *in vitro-in vivo* gap. The majority of mechanistic studies are performed in simplified 2D cell culture. The *in vivo* microenvironment is infinitely more complex, with 3D architecture, mechanical forces, and a dynamic milieu of signaling molecules. The epigenetic state and fate of an ADSC in a petri dish may not accurately reflect its behavior when transplanted into a living organism, posing a major translational challenge. Last but not the least, the profound influence of donor age, disease status, and lifestyle on the ADSC epigenome represents the single greatest challenge for creating standardized, “off-the-shelf” therapies. An epigenetic-based protocol optimized for ADSCs from a young, healthy donor may be ineffective or even harmful if applied to cells from elderly, diabetic patients. Overcoming this requires a move toward personalized therapeutic strategies.

### Tools/advances are needed to move the field forward

To address these challenges, the field requires the development and application of next-generation tools and approaches, such as single-cell multi-omics and AI-powered integrative analysis. To dissect heterogeneity and directly link epigenetic states to functional outputs, we must move beyond bulk analysis. Technologies that can simultaneously profile the transcriptome, epigenome (*e.g.*, chromatin accessibility *via* scATAC-seq), and DNA methylome from the same single cell are essential. This will allow for the construction of high-resolution maps of differentiation trajectories and the identification of the precise epigenetic events that drive fate decisions in individual cells. Additionally, the complexity of the epigenetic code, involving combinatorial patterns of dozens of modifications across the genome, is beyond human interpretation. Applying machine learning and artificial intelligence to integrate vast multi-omic datasets is necessary to identify the complex epigenetic signatures that predict cell fate and response to therapy, paving the way for the personalized medicine approaches that will be required for the successful clinical application of ADSCs.

### New insights and future directions

The discovery of “metabolic memory” in ADSCs from diabetic patients and depot-specific gene expression patterns suggests that ADSCs possess a baseline “epigenetic setpoint.” This setpoint, established by genetics, donor age, and long-term environmental exposures, may not rigidly determine cell fate but creates a biased landscape that predisposes the cells to differentiate more readily down certain pathways. A key future direction would be to define these setpoints using multi-omic signatures and investigate whether they can be therapeutically “reset”—for instance, using epigenetic drugs like 5-aza-dC to rejuvenate the osteogenic potential of ADSCs from elderly donors. Of note, emerging evidence links nutrient-sensitive modifications (such as H2B O-GlcNAcylation) and mitochondrial DNA methylation to the functional regulation of ADSCs, suggesting a deeper bidirectional crosstalk between metabolism and epigenetics. While the conventional view holds that epigenetic mechanisms control the expression of metabolic genes, an evolving hypothesis posits the reverse: that a cell’s metabolic state directly shapes its epigenetic landscape by modulating the availability of critical substrates, such as acetyl-CoA for histone acetylation, S-adenosylmethionine for DNA and histone methylation, and NAD^+^ for sirtuin activity. Testing this model could open a novel therapeutic paradigm in which ADSC fate is directed not through genetic modification but *via* metabolic conditioning.

## Conflict of interest

The authors declare that they do not have any conflicts of interest with the content of this article.
